# Disseminated histiocytoses biomarkers beyond BRAFV600E: frequent expression of PD-L1

**DOI:** 10.18632/oncotarget.4378

**Published:** 2015-06-08

**Authors:** Zoran Gatalica, Nurija Bilalovic, Juan P. Palazzo, Ryan P. Bender, Jeffrey Swensen, Sherri Z. Millis, Semir Vranic, Daniel Von Hoff, Robert J. Arceci

**Affiliations:** ^1^ Caris Life Sciences, Phoenix, Arizona, USA; ^2^ Department of Pathology, Clinical Center, University of Sarajevo, Sarajevo, Bosnia and Herzegovina; ^3^ Department of Pathology, Anatomy, and Cell Biology, Jefferson Medical College, Philadelphia, Pennsylvania, USA; ^4^ Department of Oncology, Virginia G. Piper Cancer Center at Scottsdale Healthcare/TGen, Scottsdale, Arizona, USA; ^5^ Department of Child Health, University of Arizona College of Medicine, Phoenix, Ronald Matricaria Institute of Molecular Medicine at Phoenix Children's Hospital, Phoenix, Arizona, USA

**Keywords:** histiocytoses, biomarkers, sequencing, targeted therapy, immunotherapy

## Abstract

The histiocytoses are rare tumors characterized by the primary accumulation and tissue infiltration of histiocytes and dendritic cells. Identification of the activating *BRAFV600E* mutation in Erdheim-Chester disease (ECD) and Langerhans cell histiocytosis (LCH) cases provided the basis for the treatment with BRAF and/or MEK inhibitors, but additional treatment options are needed. Twenty-four cases of neoplastic histiocytic diseases [11 extrapulmonary LCH, 4 ECD, 4 extranodal Rosai-Dorfman disease (RDD), 3 follicular dendritic cell sarcoma (FDCS), 1 histiocytic sarcoma (HS) and 1 blastic plasmacytoid dendritic cell neoplasm (BPDCN)] were analyzed using immunohistochemical and mutational analysis in search of biomarkers for targeted therapy. *BRAF V600E* mutations were detected in 4/11 LCH and 4/4 ECD cases. A pathogenic *PTEN* gene mutation and loss of PTEN protein expression were identified in the case of HS. Increased expression of PD-L1 (≥2+/≥5%) was seen in 3/4 ECD, 7/8 LCH, 3/3 FDCS and 1/1 HS, with overall 81% concordance between 2 antibodies used in the study (SP142 vs. MAB1561 clone). These results show for the first time significant expression of the PD-L1 immune checkpoint protein in these disorders, which may provide rationale for addition of immune check-point inhibitors in treatment of disseminated and/or refractory histiocytoses.

## INTRODUCTION

The proliferative histiocytoses encompass a broad spectrum of rare tumors with clinical behavior ranging from spontaneous regression to highly aggressive disease with fatal outcome [1]. They are characterized by the accumulation and tissue infiltration of immunologically active cells, including T cells, eosinophils, macrophages and dendritic cells. The histologic types include Langerhans cell histiocytosis (LCH), Rosai-Dorfman disease (RDD), Erdheim-Chester disease (ECD), follicular dendritic cell sarcoma (FDCS) and histiocytic sarcoma. In addition, blastic plasmacytoid dendritic cell neoplasm (BPDCN) shares some characteristics with plasmacytoid monocytes (dendritic cells). Identification of the activating *BRAF V600E* mutation in a subset of histiocytoses (ECD and LCH, 50-100%) has opened a new avenue for the treatment of these disorders with BRAF and MEK inhibitors [1-5]. Studies of Bubolz et al. [3] and Haroche et al. [6-7] demonstrated some efficiency of the BRAF inhibitor vemurafenib in the treatment several patients with multisystemic and refractory ECD and LCH.

The Programmed Cell Death 1 (PD-1 or CD279) protein is a T-cell co-inhibitory receptor, which upon binding of its ligand PD-L1 (CD274) expressed by tumor cells, inhibits cytokine production and cytotoxic activity of PD-1+ tumor infiltrating T-lymphocytes, facilitating tumor progression (escape phase of cancer immunoediting). The suppression of PD-L1/PD-1 interaction using specific inhibitors has shown promising effects in the treatment of several advanced cancers, most notably in melanoma, renal cell carcinoma and non-small cell lung cancer [8-10].

Because normal dendritic cells and macrophages express PD-L1 [11], we investigated its expression by neoplasms of dendritic and related histiocytic cell neoplasms.

## RESULTS

### BRAF V600E and other genes' mutations

**Figure 1:**
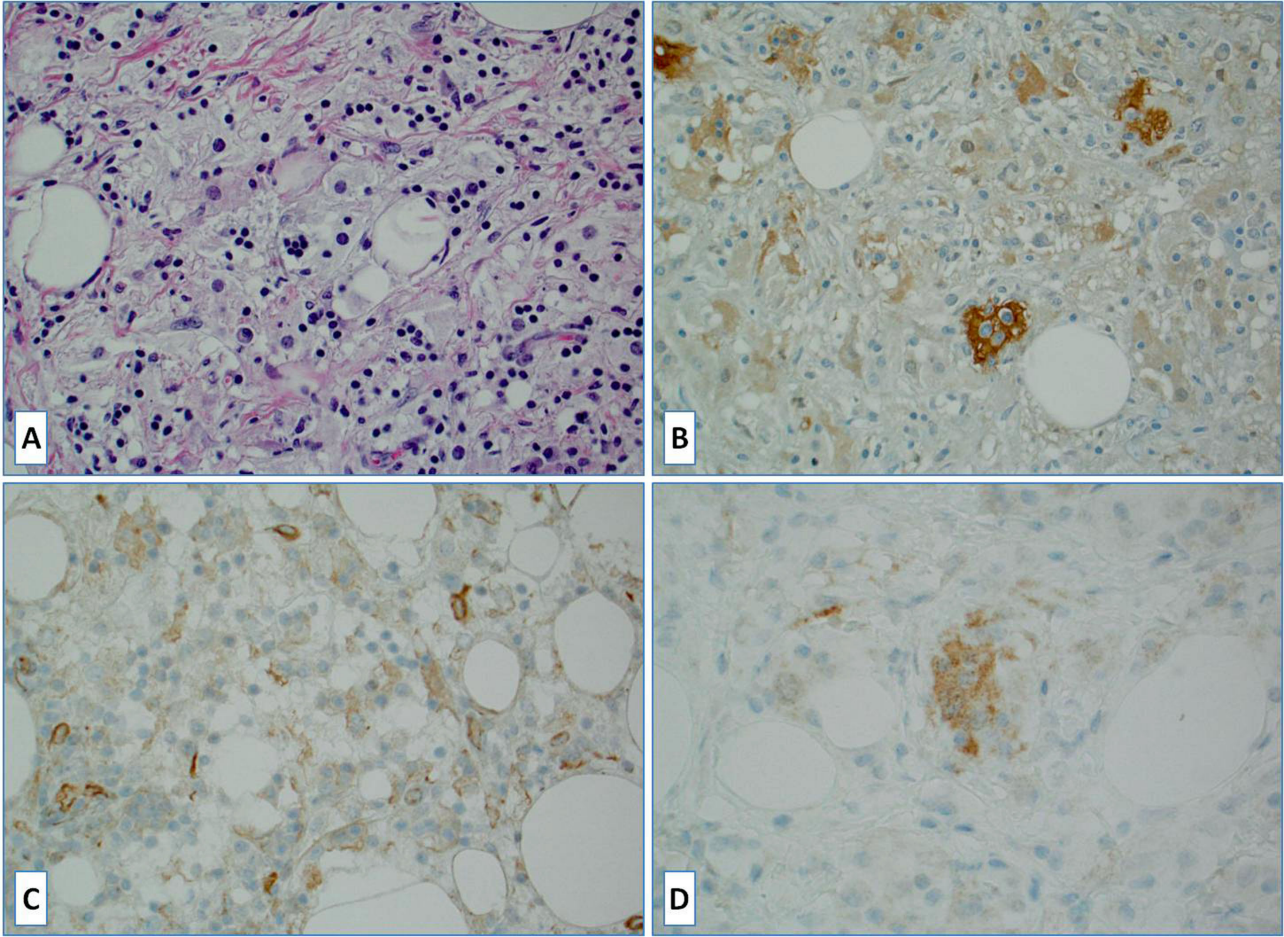
A case of Rosai-Dorfman disease **A.** Hematoxylin and Eosin [H&E] stained slide; **B.** S100 stain highlights rare disease-specific large histiocytes with emperipolesis (negative lymphocytes within S100 positive cytoplasm); **C.** Lack of PD-L1 staining in large histiocytes (scattered positive reactive cells) and **D.** BRAFV600E scattered positive large histiocytes.

**Figure 2:**
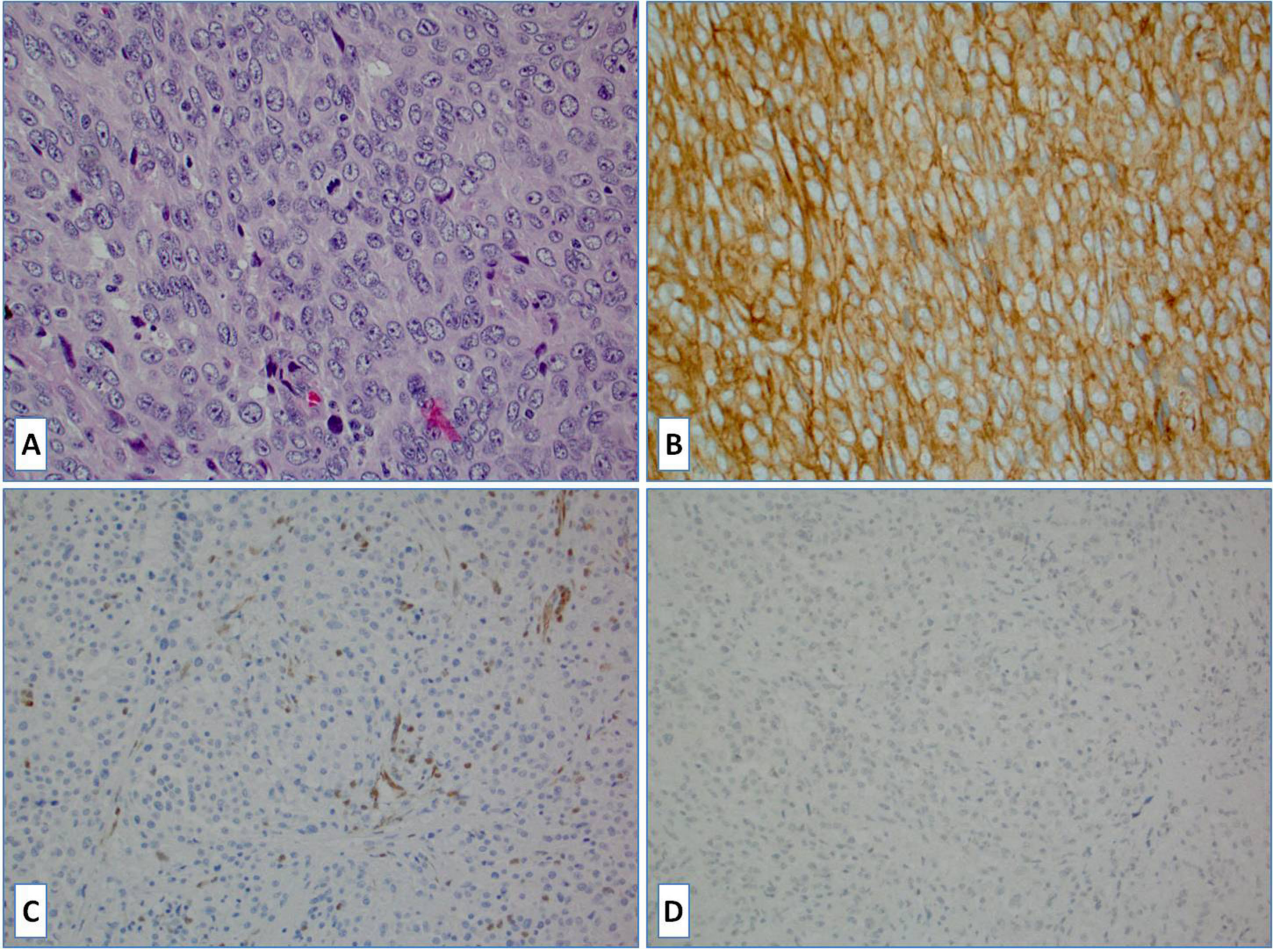
**A.** H&E slide of a case of histiocytic sarcoma; **B.** The tumor cells were strongly positive for PD-L1; **C.** The tumor completely lost PTEN protein expression due to the *PTEN* gene mutation (normal PTEN expression is seen in endothelium); **D.** No BRAFV600E mutant protein expression was observed.

**Figure 3:**
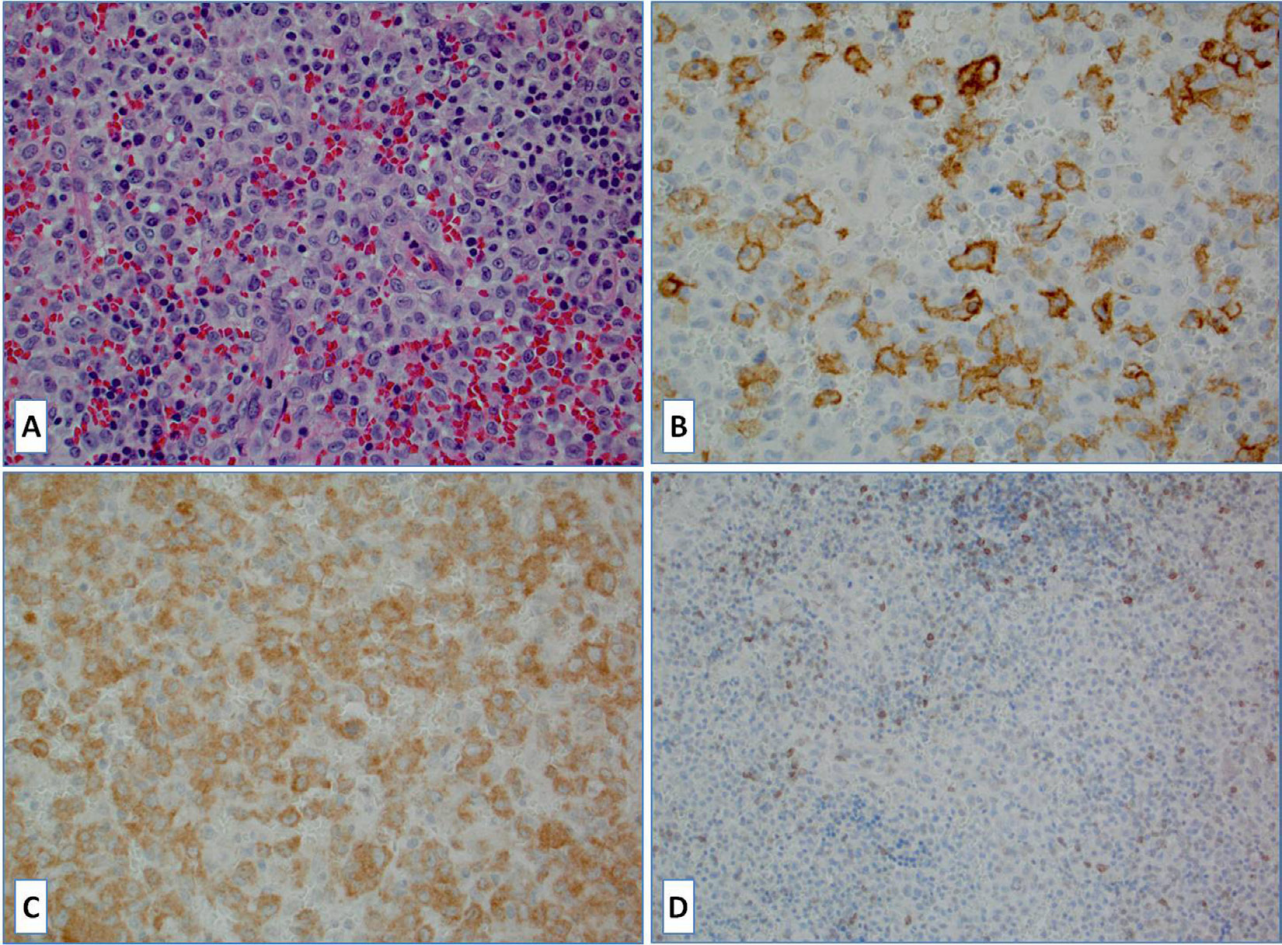
**A.** Langerhans cell histiocytosis (LCH), H&E slide; **B.** Large neoplastic Langerhans cells are strongly positive for PD-L1; **C.** BRAFV600E mutant protein expression (*BRAF* gene mutation confirmed) **D.** Tumor-infiltrating lymphocytes were positive for PD-1.

**Figure 4:**
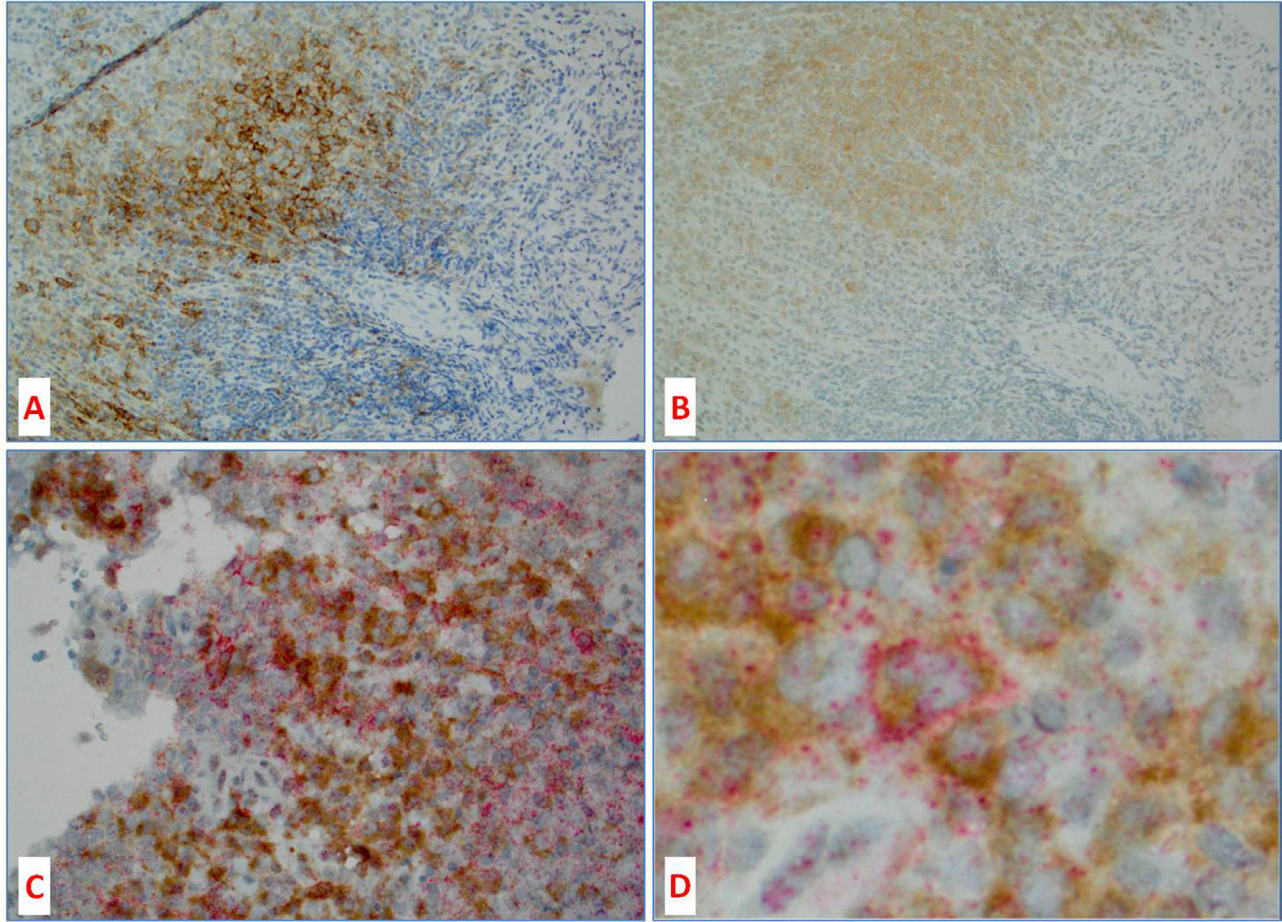
Co-localization of BRAFV600E protein and PD-L1 in histiocytic tumors **A.** PD-L1 and **B.** BRAFV600 mutant protein expression in 2 consecutive sections (8 microns apart) showing similar topographic distribution of PD-L1 and BRAFV600E; **C.** and **D.** are double IHC for PD-L1 (red) and BRAFV600E (brown) showing co-localization of both stains to the same Langerhans cell (D – center).

The *BRAF V600E* mutation was identified in 8 out of 24 cases (33%) including 4/4 ECD (100%) and 4/11 LCH (36%) while other histiocytoses harbored no *BRAF V600E* mutations (Table 1). One patient with BRAFV600E-mutated LCH involving the parietal bone harbored additional variants of unknown significance including *BRAF W604C*, *EGFR* (A743V) and *cMET* (V378I), while another patient with BRAFV600E-mutated LCH had a *JAK3* (V722I) mutation. The BRAF V600E mutant protein was detected in 3 out of 5 BRAF V600E mutated cases (60%) using immunohistochemistry (Figures 2D, 3C, 4B, and 4D) while the single BRAFV600E-sequencing-negative RDD case stained positively for BRAFV600E protein (Figure 1D). A case of HS that was devoid of a *BRAF V600E* mutation harbored pathogenic, *PTEN* mutation (c.635-7_639del; a splice site mutation that abolishes the conserved splice region at exon 7 of *PTEN* gene) confirmed by the loss of PTEN protein by IHC (Figure 2C). A variant of unknown significance involving *SMAD4* (T521I mutation) was detected in a patient with BRAF-negative extranodal RDD.

**Table 1:** Overview of BRAF, other mutations and PD-L1 status in various neoplastic histiocytoses

Histotype (n=24)	*BRAF*^V600E^ (%#)	PD-L1 expression*	Other mutations
Langerhans cell histiocytosis (n=11)	36%	88%	cMET, EGFR, BRAF W604C (1 case)** JAK3 (1 case)**
Rosai-Dorfman disease (n=4)	0%^##^	0%	SMAD4 (1 case)***
Erdheim-Chester disease (n=4)	100%	100%	None
Follicular dendritic cell sarcoma (n=3)	0%	100%	None
Histiocytic sarcoma (n=1)	0%	100%	PTEN
Blastic plasmacytoid dendritic cell neoplasm (n=1)	0%	0%	None

* 81% concordance was obtained between MAB1561 and SP142 antibodies.

** Both LCH cases also harbored BRAF V600E mutation. All described mutations represent variants of unknown significance.

*** Variant of unknown significance.

# Using COBAS method

## A single case of RDD which was negative for detection of mutations in *BRAF* (Both Cobas and NGS) was positive using IHC for mutated BRAF V600E protein.

### PD-1 and PD-L1 expression

Overexpression of PD-L1 (≥2+/≥5%) was seen in the majority of cases (3/4 ECD, 7/8 LCH, 3/3 FDCS and 1/1 HS, Table 1, Figures 1C, 2B, 3B, 4A, and 4C), but not in RDD and BPDCN. The expression of PD-L1 in neoplastic cells was appreciatively stronger (2+ and 3+ staining intensity) than in normal macrophages or dendritic cells present at the periphery of the lesions (1+). We found overall 81% concordance between two antibodies directed against PD-L1 (SP142 and MAB1561 clones). The highest concordance rate was seen in RDD (100%) and LCH (89%) while MAB1561 antibody appeared to be more sensitive in detection of positive cells in ECD (3+/3, 100%) than SP142 antibody that was positive in only 1 out of 4 tested cases (25%).

Figures 3 and 4 illustrate a case of LCH with BRAFV600E mutation expressing PD-L1 in neoplastic Langerhans cells, which in the consecutive sections show co-localization of BRAFV600E and PD-L1 positive cells. Double immunohistochemical stain also showed both PD-L1 (red) and BRAFV600E (brown) proteins co-localizing to the same multinucleated Langerhans cell.

PD-1 expressing tumor infiltrating lymphocytes were variably present in all the disease subtypes (Figure 3D).

## DISCUSSION

The histiocytoses are rare neoplasms with highly unpredictable clinical course ranging from spontaneous regression to highly aggressive disease with fatal outcome [1]. In the present study we explored the expression of the key checkpoint molecules, PD-1 and PD-L1, in these disorders along with the detection of gene mutations. The mutational analyses confirmed overall low number of mutated genes in histiocytoses [2, 15] with the pathogenic *BRAF V600E* mutations characterizing the majority of LCH and ECD [1-5]. These data support the rationale for the use of therapy targeting BRAF and/or MEK1 inhibitors for selected refractory and multisystem histiocytoses [3, 5-7]. Mutations have also been reported involving reported *KRAS, NRAS* and *PIK3CA* mutations in BRAF V600E *-*negative ECD [5, 16, 17]. We also found several additional mutations/variants of unknown significance (e.g. *cMET, EGFR*).

Although Go et al. [18] reported a high frequency of *BRAF V600E* mutations in histiocytic sarcomas, a single case in our series harbored no *BRAF V600E*; instead a pathogenic *PTEN* mutation followed by PTEN protein loss was detected. Carrasco et al. reported partial and complete *PTEN* gene deletions with concomitant loss of PTEN expression in 2 of 6 HS cases [19].

Our study for the first time demonstrates that PD-L1 frequently shows high levels of expression in systemic histiocytoses. PD-1 represents an immunosuppressive molecule whose expression on T-lymphocytes and other immune cells is an important part of immune surveillance. Activation by the ligand (PD-L1) results in attenuation of the immune response [9]. The ligand (PD-L1) may be aberrantly expressed on the neoplastic cells or on the immune cells at the tumor/host interface, and therapy-predictive contribution of either of the components still needs to be determined [8]. Inhibition of the PD-1/PD-L1 axis has been shown to be a promising therapeutic target in various cancers, particularly in renal cell carcinoma, non-small cell lung carcinoma and malignant melanoma [8-10]. We and others also reported on the expression of PD-L1 in a variety of both solid and hematological malignancies [8, 12, 20-22], but not yet in neoplastic histiocytic diseases. A study conducted by Muenst et al. [23] reported PD-1 expression in a single case of RDD. Our data indicate that a subset of histiocytoses, including LCH, ECD, FDCS, and HS, may be potentially amenable to the targeted therapy with immune checkpoint inhibitors based on the high levels of expression of these check-point molecules. This might be particularly relevant for the subset of systemic histiocytoses that are refractory to conventional and/or targeted therapies. The results also suggest the possibility of combining targeted small molecule targeted agents with PD-1/PD-L1 inhibitors. We used two different antibodies against PD-L1 to explore their diagnostic utility. Our results indicate a good concordance (81%) between the two antibodies with the MAB1561 antibody being a more sensitive than SP142 antibody. Future studies are needed to test the predictive value and utility of PD-L1 antibodies and immunohistochemistry as a method for directing targeted PD-1/PD-L1 therapy (i.e., companion diagnostic testing).

Our results of the high level of expression of PD-L1 expression in histiocytoses, along with the frequent *BRAF* and other ERK pathway gene mutations provide additional support for testing combination therapeutic approaches for patients with multisystem and/or refractory forms of histiocytoses.

## MATERIALS AND METHODS

### Samples and patients

The study included 24 patients (14 males and 10 females; age range, 2-74 years, mean: 42 years) diagnosed with histiocytic diseases including 4 cases of extranodal RDD, 11 LCH (10 extra pulmonary and 1 pulmonary), 4 ECD (2 retroperitoneal soft tissue and 2 involving cranial bones), 3 follicular dendritic cell sarcomas (FDCS, involving thorax and colon, respectively), 1 HS (bone) and 1 blastic plasmacytoid dendritic cell neoplasm (BPDCN, skin). All samples were reviewed by a board-certified pathologist (Z.G.) to confirm the diagnosis. All testing had been performed in Caris Life Sciences (Phoenix, Arizona) facility which is CLIA certified and CAP, ISO and NYSDOH accredited.

### Immunohistochemistry (IHC)

Formalin-fixed paraffin-embedded (FFPE) tissue sections were stained for PD-L1 [clones: anti-human PD-L1 rabbit monoclonal antibody S142, Spring Bioscience; and anti-human MAB1561 mouse monoclonal antibody, R&D Systems] and PD-1 (monoclonal antibody NAT105, Cell Marque). The tumor sample was considered positive for PD-L1 if 2+ intensity (complete membranous staining) was observed in ≥5% of cells [8]. Any extent of the presence of PD-1+ tumor infiltrating lymphocytes was considered positive [12]. BRAFV600E mutant protein was detected using VE1 antibody (mouse monoclonal antibody, Ventana Medical Systems, Tucson, AZ) [13, 14]. PTEN expression was analyzed using monoclonal 6H2.1 antibody (Dako, Carpinteria, CA). Selected cases were stained using double immunohistochemical staining (Figure 4) with diaminobenzidine tetra hydrochloride (DAB) as a brown chromogen for horseradish peroxidase and Naphtol and Fast red chromogen for alkaline phosphatase. Additional immunohistochemical tests were performed to confirm the diagnosis and to highlight the pathogenic cell population (e.g. S-100, CD68, CD1a detection).

### Mutational analysis

#### Next-generation sequencing (NGS)

Direct sequencing analysis was performed on genomic DNA isolated from FFPE samples using the Illumina MiSeq platform. Specific regions of the genome were amplified using the Illumina TruSeq Amplicon Cancer Hotspot panel. The panel included 46 genes sequenced by NGS and can be found at:http://www.carismolecularintelligence.com/next-generation-sequencing-profile. All variants reported by NGS are detected with >99% confidence based on the frequency of the mutation present and the amplicon coverage using a mutation frequency threshold of 10%. All regions that are sequenced achieve a minimum of 100x coverage and overall samples have an average coverage of >500x.

#### Cobas BRAF V600E analysis

*BRAF* mutation analysis was done using the C*obas*^®^ *4800 BRAF V600 Mutation Test (Roche Diagnostics)*. DNA was isolated from the FFPE tumor samples using standard laboratory procedures. Real-time PCR was used to amplify the exon 15 of the *BRAF* gene. Following amplification, a set of differentially labeled fluorescent probes were utilized to detect normal and mutant V600 sequences.
